# Study protocol for Vascular Access outcome measure for function: a vaLidation study In hemoDialysis (VALID): A multi-center, multinational validation study to assess the accuracy and feasibility of measuring vascular access function in clinical practice

**DOI:** 10.1186/s12882-022-02987-1

**Published:** 2022-11-19

**Authors:** Andrea K. Viecelli, Armando Teixeira-Pinto, Andrea Valks, Richard Baer, Roy Cherian, Pietro E. Cippà, Jonathan C. Craig, Ranil DeSilva, Allison Jaure, David W. Johnson, Charani Kiriwandeniya, Pascal Kopperschmidt, Wen-J Liu, Timmy Lee, Charmaine Lok, Krishan Madhan, Alistair R. Mallard, Veronica Oliver, Kevan R. Polkinghorne, Rob R. Quinn, Donna Reidlinger, Matthew Roberts, Bénédicte Sautenet, Lai Seong Hooi, Rob Smith, Maarten Snoeijs, Jan Tordoir, Tushar J. Vachharajani, Raymond Vanholder, Liza A. Vergara, Martin Wilkie, Bing Yang, Theodore H. Yuo, Li Zou, Carmel M. Hawley, on behalf of the VALID Investigator Team, Laura Robison, Alyssa Welch, Sunil V. Badve, Neil Boudville, Katrina Campbell, Yeoungjee Cho, Michael Collins, Magid A. Fahim, Meg Jardine, Dianne Du Toit, Michelle Mayne, Kim Stevenson, Rachel James, Quynh Vu, Karyn Allen, Leanne Glancy, Jijo Kumbikkal, Sharan Burton, Lisa Gordon, Kylee McCarthy, Cathy Forrester, Sally Lima, Olivier Bourgault, Claire Drouault, Fanny Teasdale, Liu Wen Jiun, Jamian Abidin, Cheng Jin Kiang, Lee Soon Leng, Yuana Mohd Yusoff, Adriana Ciochina, Magda van Loon, Ronald Ophelders, Marie-Jose Vleugels, Paolo Ferrari, Marie-Ève Brodeur, Davide Giunzioni, Christine Bressan Molfese, Christopher Blackwell, Louese Dunn, Laura Gillis, Barry Gray, Sarah Jenkins

**Affiliations:** 1 Department of Nephrology, Princess Alexandra Hospital, 199 Ipswich Road, Woolloongabba, QLD 4102 Australia; 2 The Translational Research Institute, Brisbane, Australia; 3 Australasian Kidney Trials Network, The University of Queensland, Brisbane, Australia; 4 Centre for Kidney Research, School of Public Health, The University of Sydney, Sydney, Australia; 5 Mater Hospital Brisbane, Brisbane, Queensland Australia; 6 Mackay Base Hospital, Mackay, Australia; 7 Division of Nephrology, Ente Ospedaliero Cantonale, Bellinzona, Switzerland; 8 Flinders University, Adelaide, Australia; 9 University of Pittsburgh, Pittsburgh, PA USA; 10 The University of Sydney, Sydney, Australia; 11 Patient Partner, Fresenius Medical Care, Schweinfurt, Germany; 12 Sultanah Aminah, Johor Bahru, Malaysia; 13 Veterans Affairs Medical Center, Birmingham, AL USA; 14 University of Toronto, Toronto, Ontario Canada; 15 Hervey Bay Hospital, Urraween, Queensland Australia; 16 Department of Nephrology, Monash Medical Centre, Monash Health, Clayton, VIC Australia; 17 Department of Medicine, Monash University, Clayton, VIC Australia; 18 Department of Epidemiology & Preventive Medicine, Monash University, Clayton, VIC Australia; 19 Departments of Medicine and Community Health Sciences, Cumming School of Medicine, University of Calgary, Calgary, Canada; 20 Eastern Health Clinical School, Monash University, Melbourne, Australia; 21 Tours University, Tours, France; 22 Patient Partner, Royal Darwin Hospital, Darwin, Australia; 23 Maastricht University Medical Center, Maastricht, Netherlands; 24 Department of Kidney Medicine, Glickman Urological & Kidney Institute, Cleveland Clinic, Cleveland, USA; 25 Cleveland Clinic Lerner College of Medicine of Case Western Reserve University, Cleveland, USA; 26 Ghent University, Ghent, Belgium; 27 Sheffield Teaching Hospitals NHS Foundation Trust, Sheffield, UK; 28 Peking University People’s Hospital, Beijing, China

**Keywords:** Hemodialysis, Vascular access, Arteriovenous fistula, Arteriovenous graft, Central venous catheter, Validation, Feasibility, Core outcome, Patient engagement, Implementation

## Abstract

**Background:**

A functioning vascular access (VA) is crucial to providing adequate hemodialysis (HD) and considered a critically important outcome by patients and healthcare professionals. A validated, patient-important outcome measure for VA function that can be easily measured in research and practice to harvest reliable and relevant evidence for informing patient-centered HD care is lacking. Vascular Access outcome measure for function: a vaLidation study In hemoDialysis (VALID) aims to assess the accuracy and feasibility of measuring a core outcome for VA function established by the international Standardized Outcomes in Nephrology (SONG) initiative.

**Methods:**

VALID is a prospective, multi-center, multinational validation study that will assess the accuracy and feasibility of measuring VA function, defined as the need for interventions to enable and maintain the use of a VA for HD.

The primary objective is to determine whether VA function can be measured accurately by clinical staff as part of routine clinical practice (Assessor 1) compared to the reference standard of documented VA procedures collected by a VA expert (Assessor 2) during a 6-month follow-up period. Secondary outcomes include feasibility and acceptability of measuring VA function and the time to, rate of, and type of VA interventions.

An estimated 612 participants will be recruited from approximately 10 dialysis units of different size, type (home-, in-center and satellite), governance (private versus public), and location (rural versus urban) across Australia, Canada, Europe, and Malaysia.

Validity will be measured by the sensitivity and specificity of the data acquisition process. The sensitivity corresponds to the proportion of correctly identified interventions by Assessor 1, among the interventions identified by Assessor 2 (reference standard). The feasibility of measuring VA function will be assessed by the average data collection time, data completeness, feasibility questionnaires and semi-structured interviews on key feasibility aspects with the assessors.

**Discussion:**

Accuracy, acceptability, and feasibility of measuring VA function as part of routine clinical practice are required to facilitate global implementation of this core outcome across all HD trials. Global use of a standardized, patient-centered outcome measure for VA function in HD research will enhance the consistency and relevance of trial evidence to guide patient-centered care.

**Trial registration:**

Clinicaltrials.gov: NCT03969225. Registered on 31st May 2019.

**Supplementary Information:**

The online version contains supplementary material available at 10.1186/s12882-022-02987-1.

## Background

Hemodialysis (HD) is the commonest treatment for patients with kidney failure worldwide and can only be provided via a functioning vascular access [1]. Establishing and maintaining a functioning access, however, is one of the greatest challenges in caring for these patients. Vascular access-related complications and interventions are associated with increased patient morbidity and mortality and healthcare costs accounting for 20–25% of annual hospital admissions in patients on HD [2–4]. From a patient’s perspective, the experience and anticipation of vascular access complications are key sources of stress and can lead to anxiety about the potential for vascular access failure [5]. Improving vascular access outcomes is therefore considered a critical priority not only by patients, but also their caregivers and health professionals [6–8].

Despite increasing numbers of vascular access trials, successful interventions to improve vascular access outcomes have been sparse and compromised by highly variable, often selectively reported outcomes of limited relevance to patients and health professionals [9, 10]. Based on a systematic review of vascular access outcomes in HD trials, vascular access function was the most frequently reported outcome, yet was described in 489 different ways with “mean access blood flow” and “number of thromboses” being the most frequently used measures. Despite efforts to standardize outcome definitions for vascular access by various working groups [11–14], only a minority of HD trials made use of these standardized definitions. For example, of the 134 patency measures reported across 64 trials, only 13% were consistent with 1 or more of the standardized definitions as proposed by national and international consortiums and societies [10]. These findings underpin the need for broader implementation of standardized, patient-important outcome measures to enhance the consistency and relevance of outcome reporting in clinical trials in HD. To address this issue, considerable efforts have been made to identify and standardize vascular access outcomes that are important to patients, their caregivers and healthcare professionals that should be reported consistently in clinical research to improve the quality, reliability and relevance of research evidence that guides patient care [15, 16]. The Standardized Outcomes in Nephrology in HD (SONG-HD) initiative identified vascular access as one of four critically important core outcome domains (along with fatigue, cardiovascular disease and mortality) for clinical trials in HD based on a consensus-based, multiphase, mixed-methods process involving over 1300 patients, caregivers and health professionals from more than 70 countries [6, 8, 17–19]. Based on international contributions of 237 patients and 720 clinicians, researchers, policy makers and industry from 58 different countries, “vascular access function” was deemed the most critically important outcome because of: 1. the broad applicability of function regardless of the vascular access type; 2. the involvement of a multidisciplinary team in achieving a functioning vascular access; and, 3. the impact of vascular access function on quality of life, survival, and other vascular access-related outcomes. “The need for interventions to enable and maintain the use of a vascular access for HD” was considered a simple, pragmatic, and inexpensive measure of vascular access function that was meaningful and relevant to patients [20]. Stakeholders considered the frequency (rate) of vascular access interventions and the intervention-free time to be key descriptors of a functioning vascular access [20]. In order to ensure global implementation of this outcome measure across all trials in HD, it needs to be feasible to accurately measure vascular access function as part of routine clinical practice without requiring additional resources or expertise in vascular access [20, 21].

While vascular access interventions have been reported in HD research and collected by renal registries [22–27] the granularity of data collection (i.e., type of vascular access interventions, date of intervention, and indication of intervention) vary substantially across studies and registries precluding reliable comparisons across trials and countries to inform research and clinical practice. A Canadian experience including five Canadian dialysis programs has shown that granular data collection of all types, dates, and indications of vascular access interventions in patients on HD using the Dialysis Measurement Analysis and Reporting (DMAR) system is feasible and reliable [22, 27, 28]. However, it remains unknown whether these data can be collected in other countries and different settings (i.e., in-center versus satellite versus home-based HD; rural versus urban; private versus public; small versus large units) by clinical staff without special expertise in vascular access and as part of routine clinical practice. VALID will address this uncertainty by assessing the validity, acceptability and feasibility of measuring vascular access function, defined by the need for interventions to enable and maintain the use of a vascular access for HD, by clinical staff as part of their routine clinical practice in a prospective, multi-center, multinational validation study covering a broad range of HD settings to ensure successful implementation of this core outcome measure in research and clinical practice without the need for additional resources or expertise in vascular access [21].

## Methods

### Aims

The primary aim of this study is to determine the accuracy of collecting vascular access interventions required to enable and maintain the use of a vascular access for HD by clinical staff as part of routine clinical practice compared to the reference standard of the documented vascular access procedures collated by a vascular access expert during a 6-month follow-up period.

Secondary aims are to assess the feasibility of this data collection process across different clinical settings and countries and to assess the accuracy of collecting details on all vascular access interventions including types of interventions.

We hypothesize that vascular access function can be measured accurately by clinical staff as part of routine clinical practice without requiring advanced vascular access and research expertise, significant time investment or costly equipment.

### Study design and setting

**Fig. 1:**
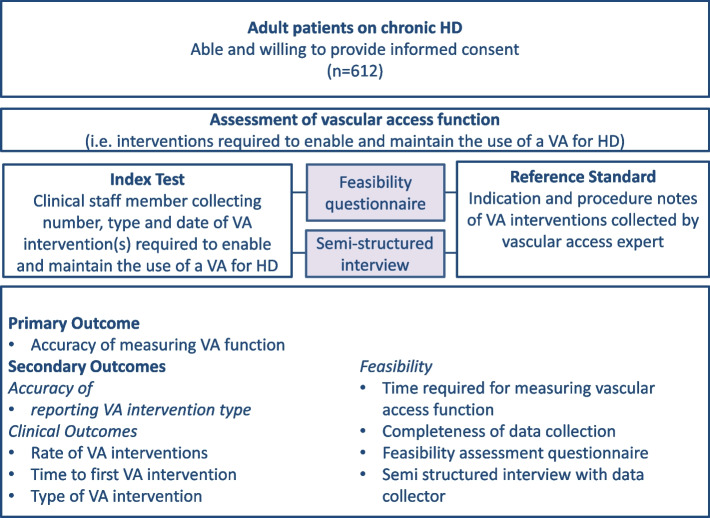
Study Schema. Abbreviations: HD – Hemodialysis; VA – Vascular Access. The study design aligns with the STARD 2015 guidelines: An Updated List of Essential Items for Reporting Diagnostic Accuracy Studies [29]

VALID is a prospective, multi-center, multinational validation study that will assess the accuracy and feasibility of measuring vascular access function in patients undergoing maintenance HD by clinical staff as part of routine clinical practice in 10 HD centers across 7 countries (Australia, Canada, France, Malaysia, the Netherlands, Switzerland, and the United Kingdom). The study schema is displayed in Fig. 1. Study sites have been purposefully selected to capture a broad range of different HD settings including geographical location (international sites, rural/remote and urban HD units), unit size (small to large HD units), HD modality (in-center-, satellite, home-based HD) and setting (private versus public hospital).

**Fig. 2:**
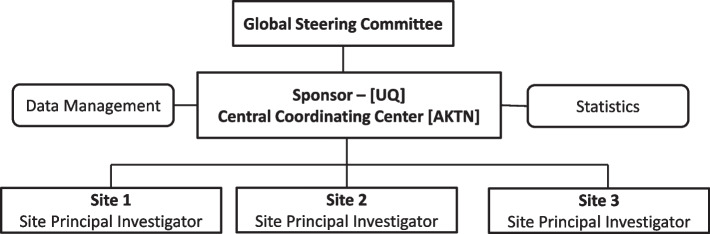
Study Governance Structure. Abbreviations: AKTN – Australasian Kidney Trials Network; UQ – University of Queensland, Australia

The study will be conducted according to International Council for Harmonization of Technical Requirements for Pharmaceuticals for Human Use (ICH) Good Clinical Practice (GCP) guidelines and will be reported according to the Standards for Reporting of Diagnostic Accuracy (STARD) guidelines [29]. The study will be conducted and coordinated by the Australasian Kidney Trials Network (AKTN), led by an International Trial Steering Committee of vascular access experts including two patient partners, study investigators, statisticians, and other researchers (Fig. 2). The VALID study protocol was registered with the ClinicalTrials.gov (NCT03969225) on 31st May 2019. Ethics Approval has been granted for all participating sites (please refer to the Declaration Section for further details). Written consent will be obtained from study participants by the site investigators in line with the site-specific ethics approval document.

### Study participants

All patients receiving maintenance HD in the participating units and who are able and willing to provide informed consent (unless a waiver of consent is approved by the local ethics committee), will be invited to take part in the trial. Patients who are expected to require HD for less than 3 months due to anticipated recovery of kidney function or are considered unsuitable to participate by clinical staff will be excluded. The eligibility criteria are deliberately kept broad to reflect routine clinical practice (i.e. not excluding non-English speaking patients) and avoid selection bias within participating units. Pediatric patients are excluded because vascular access outcomes were not considered a core outcome domain for research in pediatric patients with chronic kidney disease, as established by the SONG-Kids initiative [30].

### Test method

#### Index test

A clinical staff member (Assessor 1) of the participating HD center without special expertise in vascular access research will collect the number, type and date of any vascular access interventions required to enable and maintain the use of the participants’ vascular accesses for HD. Data will be entered in the Research Electronic Data Capture (REDCap), a secure, web-based data base application designed to support data capture for research studies and hosted by the Queensland Clinical Trials and Biostatistics Centre at the University of Queensland [31]. Assessor 1 will be, in most cases, a HD nurse working in the HD unit. Alternatively, Assessor 1 may also be a HD technician, a medical or nursing student or a junior doctor. Data collection for each patient will occur for 6 months or until the patient’s death, transfer to a different kidney replacement therapy (i.e., peritoneal dialysis, kidney transplantation), treatment withdrawal, transfer to a different unit, or kidney recovery with removal of any HD vascular access. Information on vascular access interventions will be acquired as per local practice. Resources may include (but are not limited to) electronic or paper-based medical records, existing databases, or information provided by staff or patients. Assessor 1 may prospectively enter the details on vascular access interventions into REDCap as they occur or retrospectively at 3 and 6 months of follow-up.

#### Reference standard

The reference standard represents the vascular access procedure notes (i.e., source document) collected by a vascular access expert of the research team, henceforward referred as Assessor 2. The redacted procedure notes will be uploaded into REDCap. Assessor 2 will collect the data independently from Assessor 1. Moreover, Assessor 2 will not be able to access data on vascular access interventions entered by Assessor 1 and vice versa. Assessor 2 may collect these data prospectively or retrospectively at 3 and 6 months of follow-up. This reference standard reflects the accepted research standard of collecting data on vascular access events (i.e., interventions).

#### Audit assessor role

To ensure completeness and accuracy of data collection by Assessor 2 as the gold standard, a local audit assessor at each site (typically the site principal investigator) will check completeness and verify vascular access procedure notes of 10% of randomly selected participants. In case of VA intervention missingness or inaccuracy for more than 1 participant, the audit assessor will cross-check data accuracy and completeness for all remaining study participants enrolled at the unit.

#### Maintenance of independence of assessors

In view of the importance of independence of Assessor 1 (Index test) and Assessor 2 (Reference standard), the database structure has been designed to engage role-based access control for assessors. This ensures that assessors of one category (Assessor 1, Assessor 2 or Audit Assessor) can only access the relevant sections in the database for their role, enabling them to only perform functions specified by the study protocol for that role.

### Data collection

#### Participant demographic and medical history

Table 1 summarizes the data collection timepoints for Assessor 1 and 2, respectively. Baseline data will be collected by the site investigators, typically Assessor 2, including patient demographics (sex, age, ethnicity), comorbidities (diabetes mellitus, cardiovascular diseases [ischemic heart disease, cerebrovascular accidents, peripheral vascular disease], BMI), HD and vascular access details (HD setting [in center, satellite, home HD, in center nocturnal HD, home nocturnal HD], dialysis duration, usual number of dialysis sessions per week, usual number of hours of dialysis per week, vascular access in use, location of AVF/AVG in use [upper arm, lower arm or leg], self-cannulation and cannulation technique [Rope ladder versus button hole]).

**Table 1:** Data collection schedule

Time point of data collection (months)	Assessor 1 (Index test)			Assessor 2 (Reference standard)		
	0	3	6	0	3	6
Assessor characteristics	√			√		
Patient consent (if required)				√		
Patient characteristics				√		
Number of vascular access intervention(s)		√	√		√	√
Type of vascular access intervention(s)		√	√		√	√
Date of vascular access intervention(s)		√	√		√	√
Extraction of procedure notes					√	√
Indication for vascular access intervention					√	√
Time required to collect/enter vascular access intervention data		√	√		√	√
Study end / Early exit		√^a^	√		√^a^	√
Feasibility questionnaire			√			√
Semi-structured interview			√			√

^a^Early exit from the study

Information about the roles and expertise of Assessors 1 (index test) and Assessors 2 (reference standard) will also be collected at baseline including role/profession, and years of experience in HD, in clinical research and in vascular access monitoring/maintenance.

#### Vascular access interventions

Table 2 lists all vascular access interventions considered for defining vascular access function and encompasses any interventions/procedures required to enable and maintain the use of the vascular access for HD during the study period. The selection of vascular access interventions was informed by previous research [22, 27, 28] and feedback from patients, caregivers and health professionals with expertise in vascular access [20, 32]. Interventions are considered relevant if they are performed to enable and maintain the function of the vascular access and are regarded as invasive or disruptive to the patient, i.e., potentially painful and requiring a visit to the operating room, radiology suite or special procedure room or are conducted by the bedside. To facilitate data accuracy verification, Assessor 2 will also collect the indication for each of the vascular access interventions (Supplementary Item S1).

**Table 2:** Vascular access interventions required to enable or maintain the use of a vascular access for hemodialysis

Vascular access	Intervention/Procedure
**AVF/AVG**	Open surgical or endovascular creation/placement of AVF/AVG
	Open surgical revision or endovascular intervention of AVG/AVF
	Thrombolysis or thrombectomy of AVG/AVF
	Ligation or resection of arteriovenous access
	Repair of aneurysm/pseudoaneurysm
	Competing/collateral vein ligation
	Fistulogram (Angiogram) +/− angioplasty +/− stenting (including inflow artery, body of AVF/AVG, venous outflow, central vein)
	Competing/collateral vein embolization
	Superficialization/transposition
	Management of Dialysis Associated Steal Syndrome (DASS)/Access Induced Ischemia. Procedures include:
	• Distal Revascularisation, Interval Ligation (DRIL)
	• Proximalization of the Arterial Inflow (PAI)
	• Revision Using Distal Inflow (RUDI)
	• Banding
**CVC**	CVC insertion
	CVC exchange
	Fibrin sheath removal/disruption
	CVC removal

Vascular access interventions not to be included: Repositioning of patient on HD chair/bed, reversing of CVC lines, flushes of CVC lines, repositioning of dialysis needles in AVG or AVF, thrombolytics for CVC lines, use of antibiotics for vascular access-related infections

*Abbreviations*: *AVF* Arteriovenous fistula, *AVG* Arteriovenous graft, *CVC* Central venous catheters (tunnelled and un-tunnelled)

#### Feasibility assessment

Both data collectors, Assessors 1 and 2, will be prompted to complete a feasibility questionnaire addressing all relevant feasibility aspects, as outlined by Prinsen et al. [33] at the end of study (Supplementary Item S2).

#### Semi-structured interview

At study end, face-to-face or video-call semi-structured interviews will be performed with all Assessors. The interview question guide is outlined in Supplementary Item 3. All interviews to be conducted in English will be performed by the Chief Principal Investigator AV and expected to take approximately 30 minutes. If required, interviews will be conducted in the assessor’s native language by a bilingual member of the SONG vascular access working group [32]. The interview serves to further explore assessors’ perspectives on the feasibility of measuring vascular access function. The interviews will be audio-recorded and transcribed verbatim.

#### Early withdrawal from the study

Once a participant has been included in the study, the investigator will make every reasonable effort to keep the participant in the study. Reasons that participants may be withdrawn from the study prior to study end include death, kidney transplantation, transfer to peritoneal dialysis, permanent withdrawal from kidney replacement therapy, transfer to another HD unit which is not an active study site, loss to follow-up, withdrawal of participant consent and at the discretion of the treating physician. In case of early termination of participation, the Site Principal Investigator or co-investigator (assessors 1 or 2) will complete the study end form at time of withdrawal of the participant.

### Study outcome measures

#### Primary outcome measures

The primary outcome will be the accuracy of reporting vascular access function during a 6-months period, defined by the need for any intervention(s) required to enable and maintain the use of a vascular access for HD. The accuracy of reporting vascular access function is defined by the correct identification of each intervention and the correct reporting of the total number of interventions per patient. An intervention reported by Assessor 1 will be considered correctly identified if its intervention date is within +/− 3 days of the date identified by Assessor 2 (the reference standard).

#### Secondary outcome measures

Accuracy of the reported type of VA intervention will be analyzed as secondary outcomes. Clinical outcomes include the rate of vascular access interventions (n/patient-year), the time to first vascular access intervention, and the type of vascular access interventions. Feasibility outcome measures include the time required for measuring vascular access function, completeness of data collection, Likert score of feasibility questionnaire with data collectors (Assessors 1 and 2), qualitative analysis of semi-structured interview with data collectors, and study feasibility outcomes including recruitment rate, eligibility ratio and enrolment ratio.

#### Adverse events

The study has no impact on patients’ safety, or the care provided to participants during the study conduct. No adverse events (serious or not) will be collected for this study other than the outcome measure of interest and outcomes captured as reasons for early termination of participant follow-up due to outcomes such as death or treatment withdrawal.

### Statistical considerations

#### Sample size calculation

The sample size has been calculated for the sensitivity, i.e. the proportion of interventions correctly identified by Assessor 1 and specificity, i.e. the proportion of patients with no interventions recorded by Assessor 1 that do not have a record of interventions.

Assuming an event rate of 2 vascular access interventions/ patient-year based on recent data from a study collecting a comparable range of vascular access interventions in HD patients [28], the overall expected number of interventions over a 6 month period will be equal to the number of participants, although there will be patients with no intervention and patients with more than one intervention. Assuming a true sensitivity of 90%, 520 records of interventions will provide an approximate 5% width for the 95% confidence interval.

With the rate of 2 vascular access interventions/ patient-year and assuming a Poisson distribution, the expected number of patients without an intervention in the 6 months is 193. This number will allow the calculation of the specificity with a 10% width for the 95% confidence interval, assuming a true specificity of 90%.

The sample size will be inflated to account for 15% potential dropouts (i.e. early study exit). This corresponds to a final sample size of 612 patients.

#### Accuracy and validity assessment

The unit of analysis is intervention rather than patient because each patient can have multiple interventions. Validity will be measured by the sensitivity and specificity of the data acquisition process. The sensitivity corresponds to the proportion of correctly identified interventions by Assessor 1, among the interventions identified by Assessor 2 (reference standard). The specificity is computed on the patients with no interventions (as measured by Assessor 2) by using the number of patients with no interventions identified by Assessor 1. The confidence intervals will be calculated for both sensitivity and specificity using robust standard errors, through a logistic regression fitted with a generalized estimating equation (GEE) to take into account clustering by center and patient (due to the limitations of the software, each clustering level will be considered in separate models and both confidence intervals will be reported). Additionally, the percentage of patients with the correct number of VA interventions within 6 months, as identified by Assessor 1, will be presented as a measure of accuracy, together with the Cohen’s weighted kappa as the degree of agreement for the number of VA interventions per patient, reported by the two assessors. The 95% confidence intervals for the accuracy and kappa statistics will be computed by bootstrap stratified by center.

Several subgroup analyses will be conducted to investigate variability of these measures according to patients’ and centers’ characteristics. Any tests comparing measures of validity across subgroups will be performed with a logistic model, using GEE to take into account the clustering of the data by patient and center.

Accuracy of rate of vascular access interventions, time to first intervention (allowing for imprecision of +/− 3 days) and type of vascular access will also be analyzed. For the first two continuous outcomes, the accuracy of the data will be reported as the percentage of correct information collected by Assessor 1. Finally, the accuracy of type of intervention collected by Assessor 1 will be reported as the percentage of type of intervention correctly identified and the Cohen’s kappa will be presented as a measure of agreement between the two assessors. All the measures of accuracy will be presented with 95% confidence intervals.

All data will be analyzed using R software. The script for the statistical analysis is provided as Supplementary Item S4.

#### Feasibility assessment

Time required for measuring vascular access function will be reported as study participant mean with standard deviations or median with interquartile range (IQR) depending on the distribution. Any subgroup comparison will be performed with linear models fitted with GEE with clustering by center.

The feasibility questionnaire will be analyzed quantitatively by mean, median, and percentage of assessors rating feasibility aspects as feasible (4–5 points on Likert scale) and qualitatively by thematic analysis if sufficient comments are provided.

The transcripts of the semi-structured interviews with Assessors 1 and 2 on feasibility aspects of data collection will be imported into HyperRESEARCH (ResearchWare, USA) and analyzed thematically. Thematic analysis involves identifying, examining, coding, comparing and grouping concepts to develop themes that describe the phenomenon being investigated and addresses the research question [34]. Using thematic analysis, the Chief Principal Investigator will code the transcript line-by-line to identify concepts relevant to the assessors’ perspectives on the feasibility of measuring vascular access function as part of routine clinical practice and for research purposes. The preliminary codes will then be reviewed by co-investigators who will independently read the transcripts and discuss any feedback with the chief investigator. This form of investigator triangulation can enhance the analytical framework and ensure that the full range and depth of data are captured in the initial analysis.

### Data management and quality assurance

#### Data handling and record retention

The VALID study data will be captured and stored electronically via REDCap. Original consent forms will be stored locally. After closure of the trial, investigators will maintain all study documentation, including consent documents, ethics committee approvals and correspondence, for a minimum of 15 years, or as per local guidelines.

#### Data sharing

Data sets will be made available by the Central Coordinating Group to researchers within the VALID study for analysis of sub-studies and country specific outcomes after the primary manuscript has been accepted for publication.

For researchers outside the VALID study, individual participant data will be made available upon request to a Data Access Committee, a review board set up to assess proposals based on sound science, benefit-risk balancing and research team expertise. Appropriate data will be made available to approved proposals. This process will be in effect for a period of 2 to 5 years following publication of the main study results. After 5 years, the data will be available in the Sponsor’s data warehouse but without investigator support other than deposited metadata.

#### Training

All data collectors will receive an induction to using REDCap. An operational user manual for REDCap will also be provided to the participating units. Site Principal Investigators and their co-investigators will also have to meet the following criteria: Adequate time to conduct the study, adequate training and experience to conduct the study, ability to recruit enough participants to conduct the study, and provide evidence of proficiency in the tenets of Good Clinical Practice.

#### Central monitoring

Utilizing a risk-based monitoring approach, a detailed monitoring plan will outline trial monitoring activities. Monitoring efficiency will be optimized by a system of remote monitoring performed by the Central Coordinating Group of the AKTN. If indicated, and with advance notice, study sites may be visited by a Clinical Monitor. The visits will be an opportunity to provide additional support and training to site staff, ensure the study is conducted according to the protocol, and in line with local regulatory requirements. Source documents from which the data are obtained will be made available by the site for review.

#### Data quality assessment

The adjudication committee (Audit assessors) will inspect the source data against recorded data collected by Assessor 2 for completeness and accuracy as described under the Audit assessor role.

### Ethical consideration and dissemination

#### Modification of the protocol

Any modifications to the protocol which may impact on the conduct of the study including changes of study objectives, study design, study population, sample size, study procedures, or significant administrative aspects may require a formal amendment to the protocol. Such amendment will be agreed upon by the global Trial Steering Committee, and approved by the Independent Ethics Committee prior to implementation and notified to the health authorities in accordance with local regulations.

#### Early termination of study

The study will continue to its planned end unless it is no longer practicable to complete the study, either overall or at an individual site. If such action is taken, the reasons for terminating the trial will be documented in detail. All trial subjects still under follow-up at the time of termination will undergo a final assessment. The Chief Principal Investigator and the Central Coordinating Group must be informed without delay if any investigator has any concerns about continuation of the trial.

#### Protection of participant confidentiality

Participants’ records and the data generated by the study will be confidential. Any information that may identify a participant will be excluded from data presented in the public arena. Data will be stored in a secure, lockable location, and access to electronic data will be protected through a password protected web interface. The data extracted will be de-identified and a unique study number used. Similarly, data collected on the electronic case report form will be de-identified and a unique subject number will be used.

#### Dissemination of study outcomes

Study results will be disseminated via publications in peer-reviewed journals, presentations at national and international scientific meetings, and social media as well as State Renal Networks, the SONG initiative database and the AKTN website.

## Discussion

Vascular access function has been identified as one of the most critically important outcome measures for trials in HD that is pragmatic and meaningful [20, 35], yet, this outcome has not been reported consistently across clinical trials [10]. The VALID study will address this issue by assessing the accuracy, acceptability and feasibility of measuring vascular access function, defined by any vascular access intervention required to enable and maintain HD vascular access function, in an international validation study that covers a broad range of different HD settings to ensure successful implementation of this core outcome measure in research and clinical practice without the need for additional resources or expertise in vascular access [21].

VALID follows the three key strategies for successfully implementing core outcomes in research. Firstly, all relevant stakeholders, including patients, have been engaged in the identification of the core outcome and development process of the study [6, 8, 14, 17, 19, 20, 35]. Secondly, feasibility and validity of proposed core outcomes will be demonstrated and is the basis for the protocol in this manuscript. Thirdly, the reasons for, and type of core outcomes will be disseminated globally [33]. Global implementation of a validated outcome measure for vascular access function in HD trials, national kidney registries, and clinical practice would be a major step forward in reporting what is important to patients and will facilitate quality improvement as well as maximize the chances of discovering effective interventions to reduce the burden and cost of vascular access interventions required to maintain the function of the patients’ vascular accesses, their lifelines for HD.

The strength of this research is that it uses a robust, adequately powered methodological approach to evaluate the accuracy and feasibility of measuring vascular access function in various clinical setting and locations. The study has been developed and designed by patients on HD and vascular access experts (nephrologists, nurses and vascular access surgeons), researchers and statisticians with established track records of excellence in the conduct of large-scale clinical trials. The study has considerable buy-in from dialysis units in Australia, Europe, Canada, and Malaysia and will be overseen internationally by the Australasian Kidney Trials Network, which has a strong track record in adopting novel, pragmatic trial designs and generating new knowledge that changes practice in kidney failure care. The broad range of clinical settings in which this study will be conducted, such as small and large centers, rural and urban areas, in-center, satellite and home-dialysis facilities, public and private sectors, will enhance the external validity of this study. Importantly, the study adheres to the STARD diagnostic accuracy tests guidelines. The limitations include the potential for incorrect or missing vascular access data collated by Assessor 2 serving as the reference standard: however, the audit assessor double checking data accuracy should mitigate this risk. Furthermore, the study does not include centers from low- to very-low-income countries thereby limiting the applicability of study findings to these settings. Independence of data acquisition between Assessor 1 and 2 could potentially be breached by assessors thereby impacting the accuracy of study findings. Several risk mitigation strategies have been put in place including enforcement of independence in the trial operations manual and protocol, a special emphasis during the site initiation visit and individual database access with restricted view of the data by assessors.

In summary, the VALID study will evaluate the accuracy, acceptability and feasibility of measuring vascular access function as part of routine clinical practice, as well as determine the frequency of vascular access interventions across different HD settings and countries. This will facilitate global implementation of this patient-important core outcome measure in HD trials to improve the quality and relevance of research to inform patient-centered care and reduce research waste.

### Trial status

The current VALID protocol is Version 1.1 dated 5th August 2019. Recruitment commenced on 9th December 2019 and concluded on 30th November 2021. The study including participant follow-up, feasibility assessment and auditing of data accuracy by the Audit assessors is estimated to be completed by December 2022 when the dataset will be locked. The study protocol was submitted after recruitment of all sites was completed due to earlier than anticipated achievement of the target sample size and enrollment of all participants within a site within 4 weeks of site initiation.

## Supplementary Information

**Additional file 1:** **Supplementary Item S1**. Indication for Vascular Access Intervention. **Supplementary Item S2.** Feasibility Assessment Questionnaire. Feasibility assessment questionnaire adapted from Prinsen et al. *Trials*, 2016;17:449. **Supplementary Item S3.** Semi-Structured Interview Guide. Semi-structured interview guide to describe the assessors’ perspectives on the feasibility of measuring vascular access function (adapted from Prinsen et al. *Trials*, 2016;17:449). **Supplementary Item S4.** Statistical Analysis Plan using R software.

## Data Availability

The datasets used and/or analyzed during the current study are available from the corresponding author on reasonable request. The statistical analysis plan has been attached as Supplementary Item 4.

## Abbreviations

AKTN: Australasian Kidney Trials Network
AVF: Arteriovenous Fistula
AVG: Arteriovenous Graft
CVC: Central Venous Catheter
DMAR: Dialysis Measurement Analysis and Reporting
GCP: Good Clinical Practice
HD: Hemodialysis
ICH: International Council for Harmonization
REDCap: Research Electronic Data Capture
SONG: Standardized Outcomes in Nephrology
STARD: Standards for Reporting of Diagnostic Accuracy
VALID: Vascular Access outcome measure for function: a vaLidation study In hemoDialysis

## References

[1] Liyanage T, Ninomiya T, Jha V, Neal B, Patrice HM, Okpechi I (2015). Worldwide access to treatment for end-stage kidney disease: a systematic review. Lancet..

[2] Hakim R, Himmelfarb J (1998). Hemodialysis access failure: a call to action. Kidney Int.

[3] Arora P, Kausz AT, Obrador GT, Ruthazer R, Khan S, Jenuleson CS (2000). Hospital utilization among chronic dialysis patients. J Am Soc Nephrol.

[4] Manns B, Tonelli M, Yilmaz S, Lee H, Laupland K, Klarenbach S (2005). Establishment and maintenance of vascular access in incident hemodialysis patients: a prospective cost analysis. J Am Soc Nephrol.

[5] Taylor MJ, Hanson CS, Casey JR, Craig JC, Harris D, Tong A (2015). "You know your own fistula, it becomes a part of you"-Patient perspectives on vascular access: A semistructured interview study. Hemodial Int.

[6] Tong A, Manns B, Hemmelgarn B, Wheeler DC, Tugwell P, Winkelmayer WC (2015). Standardised outcomes in nephrology - Haemodialysis (SONG-HD): study protocol for establishing a core outcome set in haemodialysis. Trials..

[7] Manns B, Hemmelgarn B, Lillie E, Dip SC, Cyr A, Gladish M (2014). Setting research priorities for patients on or nearing dialysis. Clin J Am Soc Nephrol.

[8] Tong A, Manns B, Hemmelgarn B, Wheeler DC, Evangelidis N, Tugwell P (2017). Establishing core outcome domains in hemodialysis: report of the Standardised Outcomes in Nephrology – Hemodialysis (SONG-HD) consensus workshops. Am J Kidney Dis.

[9] Al-Jaishi AA, Liu AR, Lok CE, Zhang JC, Moist LM (2017). Complications of the arteriovenous fistula: a systematic review. J Am Soc Nephrol.

[10] Viecelli AK, O'Lone E, Sautenet B, Craig JC, Tong A, Chemla E (2018). Vascular access outcomes reported in maintenance hemodialysis trials: a systematic review. Am J Kidney Dis.

[11] Lee T, Mokrzycki M, Moist L, Maya I, Vazquez M, Lok CE (2011). Standardized definitions for hemodialysis vascular access. Semin Dial.

[12] Sidawy AN, Gray R, Besarab A, Henry M, Ascher E, Silva M (2002). Recommended standards for reports dealing with arteriovenous hemodialysis accesses. J Vasc Surg.

[13] Gray RJ, Sacks D, Martin LG, Trerotola SO (2003). Society of interventional radiology technology assessment C. Reporting standards for percutaneous interventions in dialysis access. J Vasc Interv Radiol.

[14] Vascular Access Working Group (2006). Clinical practice guidelines for vascular access. Am J Kidney Dis.

[15] Standardised Outcomes in Nephrology (SONG) Initiative. Available from: https://songinitiative.org/. Accessed 12 Apr 2022.

[16] Shenoy S, Allon M, Beathard G, Brouwer-Maier D, Dember LM, Glickman M (2018). Clinical trial end points for hemodialysis vascular access: background, rationale, and definitions. Clin J Am Soc Nephrol.

[17] Urquhart-Secord R, Craig JC, Hemmelgarn B, Tam-Tham H, Manns B, Howell M (2016). patient and caregiver priorities for outcomes in hemodialysis: an International nominal group technique study. Am J Kidney Dis.

[18] Sautenet B, Tong A, Williams G, Hemmelgarn BR, Manns B, Wheeler DC (2018). Scope and consistency of outcomes reported in randomized trials conducted in adults receiving hemodialysis: a systematic review. Am J Kidney Dis.

[19] Evangelidis N, Tong A, Manns B, Hemmelgarn B, Wheeler DC, Tugwell P (2017). Developing a set of core outcomes for trials in hemodialysis: an International Delphi survey. Am J Kidney Dis.

[20] Viecelli AK, Tong A, O'Lone E, Ju A, Hanson CS, Sautenet B (2018). Report of the Standardized Outcomes in Nephrology-Hemodialysis (SONG-HD) consensus workshop on establishing a core outcome measure for hemodialysis vascular access. Am J Kidney Dis.

[21] Tong A, Manns B, Wang AYM, Hemmelgarn B, Wheeler DC, Gill J (2018). Implementing core outcomes in kidney disease: report of the Standardized Outcomes in Nephrology (SONG) implementation workshop. Kidney Int.

[22] Manera KE, Tong A, Craig JC, Brown EA, Brunier G, Dong J (2017). Standardized Outcomes in Nephrology-Peritoneal Dialysis (SONG-PD): study protocol for establishing a core outcome set in PD. Perit Dial Int.

[23] Viecelli AK, Pascoe E, Polkinghorne KR, Hawley C, Paul-Brent PA, Badve SV (2015). The Omega-3 fatty acids (Fish Oils) and Aspirin in Vascular access OUtcomes in REnal Disease (FAVOURED) study: the updated final trial protocol and rationale of post-initiation trial modifications. BMC Nephrol.

[24] Lok CE, Sontrop JM, Tomlinson G, Rajan D, Cattral M, Oreopoulos G (2013). Cumulative patency of contemporary fistulas versus grafts (2000-2010). Clin J Am Soc Nephrol.

[25] Lok CE, Moist L, Hemmelgarn BR, Tonelli M, Vazquez MA, Dorval M (2012). Effect of fish oil supplementation on graft patency and cardiovascular events among patients with new synthetic arteriovenous hemodialysis grafts: a randomized controlled trial. JAMA..

[26] Standardised Outcomes in Nephrology (SONG) Initiative. SONG Handbook (Version 1, 1st June 2017) for establishing and implementing core outcomes in chronic kidney disease. Available from: http://songinitiative.org/reports-and-publications/. Accessed 12 Apr 2022.

[27] Oliver MJ, Verrelli M, Zacharias JM, Blake PG, Garg AX, Johnson JF (2012). Choosing peritoneal dialysis reduces the risk of invasive access interventions. Nephrol Dial Transplant.

[28] Murad MH, Swiglo BA, Sidawy AN, Ascher E, Montori VM (2008). Methodology for clinical practice guidelines for the management of arteriovenous access. J Vasc Surg.

[29] Enhancing the QUAlity and Transparency Of health Research (EQUATOR network). STARD 2015: An Updated List of Essential Items for Reporting Diagnostic Accuracy Studies Available from: http://www.equator-network.org/reporting-guidelines/stard/. Accessed 12 Apr 2022.

[30] Hanson CS, Craig JC, Logeman C, Sinha A, Dart A, Eddy AA (2020). Establishing core outcome domains in pediatric kidney disease: report of the Standardized Outcomes in Nephrology-Children and Adolescents (SONG-KIDS) consensus workshops. Kidney Int.

[31] Harris PA, Taylor R, Thielke R, Payne J, Gonzalez N, Conde JG (2009). Research electronic data capture (REDCap)--a metadata-driven methodology and workflow process for providing translational research informatics support. J Biomed Inform.

[32] Standardised Outcomes in Nephrology (SONG) Initiative. SONG-Haemodialysis Vascular Access Expert Working Group Available from: http://songinitiative.org/projects/song-hd/song-hd-vascular-access/. Accessed 12 Apr 2022.

[33] Prinsen CA, Vohra S, Rose MR, Boers M, Tugwell P, Clarke M (2016). How to select outcome measurement instruments for outcomes included in a "Core Outcome Set" - a practical guideline. Trials..

[34] Braun V, Clarke V (2006). Using thematic analysis in psychology. Qual Res Psychol.

[35] Viecelli AK, Howell M, Tong A, Teixeira-Pinto A, O'Lone E, Ju A (2020). Identifying critically important vascular access outcomes for trials in haemodialysis: an international survey with patients, caregivers and health professionals. Nephrol Dial Transplant.

