# Supplementary material for: Study protocol for Vascular Access outcome measure for function: a vaLidation study In hemoDialysis (VALID): A multi-center, multinational validation study to assess the accuracy and feasibility of measuring vascular access function in clinical practice
Source: BMC Nephrol. 2022 Nov 19;23:372. doi: 10.1186/s12882-022-02987-1 (PMC9675211; doi:10.1186/s12882-022-02987-1)
Supplement: Supplementary file 1 — Additional file 1: Supplementary Item S1. Indication for Vascular Access Intervention. Supplementary Item S2. Feasibility Assessment Questionnaire. Feasibility assessment questionnaire adapted from Prinsen et al. Trials, 2016;17:449. Supplementary Item S3. Semi-Structured Interview Guide. Semi-structured interview guide to describe the assessors’ perspectives on the feasibility of measuring vascular access function (adapted from Prinsen et al. Trials, 2016;17:449). Supplementary Item S4. Statistical Analysis Plan using R software. [file 12882_2022_2987_MOESM1_ESM.docx]

**Supplementary Material**

**Supplementary Item S1. Indication for Vascular Access Intervention**

**Clinical exam and patient indicators**

Arm swelling

Chest/breast swelling

Difficult/painful cannulation

Prolonged bleeding after decannulation

Poor maturation of AVF/AVG

Decreased intra-access flow

High intra-access flow

High output congestive heart failure

Steal syndrome/ischemia

Aneurysm/pseudoaneurysm

Access-related infection (local or bloodstream)

Withdrawal of HD/transfer to PD/transplantation

Patient preference

**Dialysis indicators**

Increased venous (outlet) pressure

Worsening negative arterial (inlet) pressure

Inadequate clearance (in the absence of other causes (non-vascular access))

Increased recirculation

**Radiologic indicators**

Central venous stenosis/thrombosis

Atrial thrombosis

Vascular access thrombosis

**Mechanical indicators**

Removal of CVC

Replacement of CVC

Extruded CVC cuff/CVC pulled/fell out

Cracked hub

**Supplementary Item S2. Feasibility Assessment Questionnaire**

Feasibility assessment questionnaire adapted from Prinsen et al. *Trials*, 2016;17:449

| **Feasibility criteria** | **Response** | **Comments** |
| --- | --- | --- |
| I was able to understand the outcome measure. | - Strongly agree - Agree - Neither agree or disagree - Disagree - Strongly disagree - NOT COMPLETED |  |
| The measure was quick to report. | - Strongly agree - Agree - Neither agree or disagree - Disagree - Strongly disagree - NOT COMPLETED |  |
| It was easy to collect the *number* of vascular access interventions. | - Strongly agree - Agree - Neither agree or disagree - Disagree - Strongly disagree - NOT COMPLETED |  |
| It was easy to collect the *type* of vascular access intervention(s). | - Strongly agree - Agree - Neither agree or disagree - Disagree - Strongly disagree - NOT COMPLETED |  |
| It was easy to collect the *date* of the vascular access intervention(s). | - Strongly agree - Agree - Neither agree or disagree - Disagree - Strongly disagree - NOT COMPLETED |  |
| I would have measured and reported the outcome as part of usual clinical care. | - Strongly agree - Agree - Neither agree or disagree - Disagree - Strongly disagree - NOT COMPLETED |  |
| It was easy to apply the outcome to different patients (i.e. easy to standardize). | - Strongly agree - Agree - Neither agree or disagree - Disagree - Strongly disagree - NOT COMPLETED |  |
| Collection and data entry of vascular access intervention type and date should be performed | - Prospectively (real-time, i.e. at the time the intervention occurs) - Retrospectively every ___ weeks |  |

Note: NOT COMPLETED in this context means the assessor did not answer the question posed.

**Supplementary Item S3. Semi-Structured Interview Guide**

Semi-structured interview guide to describe the assessors’ perspectives on the feasibility of measuring vascular access function (adapted from Prinsen et al. *Trials*, 2016;17:449).

**Questions:**

1. How did you go with collecting and entering the data for this study?
2. What were the most challenging aspects of collecting and entering the data? Would you have any suggestions on how to change/improve this?
3. Did you involve the patients in gathering the data? If so, what information were they providing (i.e. whether, when, and what type of vascular access interventions they have had) and did you cross-check the accuracy of this information?
4. In your unit, do you routinely collect these data? If so, are there any differences in the way you collected these data for this study (prompts: date of the intervention, type of intervention, indication for intervention)?
5. Why did this data collection require/not require extra time? Would you have any suggestions on how to make the data collection more efficient/faster?
6. Was there any cost involved to collect the data? If so, what for?
7. Do you think the “*rate* of intervention required to enable or maintain the use of a vascular access for HD” is a meaningful way to assess vascular access function? For example, patient A required 3 interventions within a year to keep the access working compared to patient B who did not require any interventions. Why or why not? Would you have alternative suggestions?
8. Do you think measuring vascular access function as “*time to the first intervention* to enable or maintain the use of a vascular access for HD” would be more meaningful than measuring the rate of interventions? Why or why not?
9. Would you be prepared/willing to continue collecting these data for research (e.g. clinical trials) conducted in patients on HD in your unit – why/why not?
10. Would you be prepared/willing to continue collecting these data as part of your routine clinical practice (i.e. not just for research purposes) – why/why not?

**Supplementary Item S4. Statistical Analysis Plan using R software**

---

title: "SAP- VALID"

author: "Armando Teixeira-Pinto"

date: "11/04/2022"

output:

  html_document:

    toc: true

    toc_float: true

    theme: paper

editor_options:

  chunk_output_type: console

---

The R code included in this markdown document imports, formats and analyses

the data from the VALID study. Only the main analysis, evaluating the validity

of  the information collected regarding vascular access, in included.  Subgroup

and sensitivity analyses are not included in the current version.

```{r setup, include=FALSE}

set.seed(2001)

knitr::opts_chunk$set(echo = TRUE)

library(knitr)

library(kableExtra)

library(tableone)

library(ggplot2)

library(ggridges)

library(dplyr)

library(plyr)

library(irr)

library(sqldf)

library(PropCIs)

library(epiR)

library(ggplot2)

library(tidyverse)

library(lubridate)

library(crosstable)

library(geepack)

library(DescTools)

library(ggeffects)

theme_set(theme_ridges())

###Functions

#computes the weighted kappa in a

#bootstrap sample

kappa2.bootstrap <- function(data.agree,i, wght){

  kappa2(data.agree[sample(nrow(data.agree), replace=T),],

         weight=wght)$value

}

#computes the bootstrap 95%CI for the

# weighted kappa using nbt bootsample

kappa2.bootstrap.ci <- function(data.agree, nbt, wght = "squared"){

  boot.sample <- sapply(1:nbt,

                        kappa2.bootstrap,

                        data.agree=data.agree,

                        wght = wght)

  quantile(boot.sample, c(.025, .975))

}

```

# Data preparation

Data was exported from RedCap in several files. Below, we

import, clean and merge the dataset.

## Read the data

```{r include=TRUE}

###############################

#Dataset Preparation          #

###############################

setwd("/../")

#data patients characteristics

  data.pc <- read_csv("VALID-PatientCharacteristi_DATA_2020-10-08_1010.csv") %>%

                        filter(.,patient_characteristics_complete==2)

#data screen

   data.screen <- read_csv("VALID-ScreeningAndEnrolmen_DATA_2020-10-07_1542.csv")

#Date interventions by assessors 1 and 2

#Assessor 1 - checks if the data exists in the data.pc

dfa1 <- read_csv("VALID-VAInterventions_a1_DATA_2020-10-08_1013.csv") %>%

             #checks if the data exists in the data.pc

             filter(validid_start %in% data.pc$validid_start) %>%

             #Removes entries with empty intervention recorded

             filter(!is.na(hasinterventions_a1va)) %>%

             #selects the vars collected by Assessor 1

             select(., c("validid_start",

                            "hasinterventions_a1va",

                            "intervent_a1va",

                            "date_intervent_a1va",

                            "timeminutes_a1va")) %>%

             #converts the date of intervention

             mutate(date_intervent_a1va =

                      as.POSIXct(date_intervent_a1va,

                                format="%e/%m/%Y")) %>%

             #window of matching the date of intervention with assessor 2

             #plus minus 3 days

             mutate(date_lim_min = date_intervent_a1va - days(3),

                    date_lim_max = date_intervent_a1va + days(3)) %>%

            dplyr::rename("ID_A1"=validid_start)

#Assessor 2 - checks if the data exists in the data.pc

dfa2 <- read_csv("VALID-VAInterventions_a2_DATA_2020-10-08_1017.csv") %>%

              #checks if the data exists in the data.pc

              filter(validid_start %in% data.pc$validid_start) %>%

              #Removes entries with #no intervention #recorded

              filter(!is.na(hasintervention_a2va)) %>%

              #selects the vars collected by Assessor 2

              select(c("validid_start",

                          "hasintervention_a2va",

                          "intervent_a2va",

                          "date_intervent_a2va",

                          "timeminutes_a2va")) %>%

              #converts the date of intervention

              mutate(date_intervent_a2va =

                     as.POSIXct(date_intervent_a2va,

                                format="%e/%m/%Y")) %>%

              dplyr::rename("ID_A2"=validid_start)

  #creates an id for the no interventions with patient

  # (this will allow matching the no interventions)

  dfa1 <- dfa1 %>%

          filter(hasinterventions_a1va==0) %>%

          group_by(ID_A1) %>%

          dplyr::mutate(IDforMatch1 = sequence(n())) %>%

          rbind(dfa1[dfa1$hasinterventions_a1va==1,])

  #creates an id for the no interventions with patient

  # (this will allow matching the no interventions)

  dfa2 <- dfa2 %>%

          filter(hasintervention_a2va==0) %>%

          group_by(ID_A2) %>%

          dplyr::mutate(IDforMatch2 = sequence(n())) %>%

          rbind(dfa2[dfa2$hasintervention_a2va==1,])

  # Matching "no intervention"

  # for assessors 1 and 2

  data.as_temp0 <- sqldf("SELECT * FROM dfa2 LEFT JOIN dfa1 ON

                         (dfa2.ID_A2 = dfa1.ID_A1 AND

                         dfa2.IDforMatch2 = dfa1.IDforMatch1 ) WHERE

                         dfa2.hasintervention_a2va=0")

  # matching "interventions"

  # for assessors 1 and 2

  # the interventions detected by assessor 2 but not 1

  # will be included

  data.as_temp1.1 <- sqldf("SELECT * FROM dfa2 LEFT JOIN dfa1 ON

                         (dfa2.ID_A2 = dfa1.ID_A1 AND

                         dfa2.date_intervent_a2va BETWEEN

                         dfa1.date_lim_min AND dfa1.date_lim_max ) WHERE

                         dfa2.hasintervention_a2va=1  ")

  # matching "interventions"

  # for assessors 1 and 2

  # the interventions detected by assessor 1 but not 2

  # will be included

  data.as_temp1.2<- sqldf(" SELECT * FROM dfa1 LEFT JOIN dfa2 ON

                         (dfa2.ID_A2 = dfa1.ID_A1 AND

                         dfa2.date_intervent_a2va BETWEEN

                         dfa1.date_lim_min AND dfa1.date_lim_max ) WHERE

                         dfa1.hasinterventions_a1va=1")

  # merges  data.as_temp1.1 and  data.as_temp1.2

  # and removes duplicate rows

  data.as_temp1  <- rbind(data.as_temp1.1,

                           data.as_temp1.2) %>%

                      distinct()

 ####################################################################

#Main dataset with each intervention collected by assessors 1 and 2

####################################################################

data.as <- rbind(data.as_temp0,data.as_temp1) %>%  tibble()  %>%

   mutate(hasinterventions_a1va =

            ifelse(is.na(hasinterventions_a1va),

                   0, hasinterventions_a1va),

          hasintervention_a2va =

            ifelse(is.na(hasintervention_a2va),

                   0, hasintervention_a2va),

          ID_A1 = ifelse(is.na(ID_A1), ID_A2, ID_A1),

          ID_A2 = ifelse(is.na(ID_A2), ID_A1, ID_A2) ) %>%

   mutate(ID = as.numeric(factor(ID_A1))) %>%

   mutate(intervent_a2va=ifelse(intervent_a2va==1,

                               "Open surgical or endovascular

                               creation/placement of AVF/AVG",

                         ifelse(intervent_a2va==2,

                              "Open surgical revision or

                              endovascular intervention

                              of AVG/AVF",

                         ifelse(intervent_a2va==3,

                              "Thrombolysis or thrombectomy of AVG/AVF",

                         ifelse(intervent_a2va==4,

                              "Ligation or resection of Arteriovenous access",

                         ifelse(intervent_a2va==5,

                              "Repair of aneurysm/pseudoaneurysm",

                         ifelse(intervent_a2va==6,

                              "Competing/collateral vein ligation",

                         ifelse(intervent_a2va==7,

                              "Fistulogram (Angiogram) +/- Angioplasty +/-

                              Stenting (including inflow artery,

                              body of AVF/AVG, venous outflow, central vein) ",

                         ifelse(intervent_a2va==8,

                              "Competing/collateral vein embolisation",

                         ifelse(intervent_a2va==9,

                              "Superficialisation/transposition",

                         ifelse(intervent_a2va==10,

                              "Distal Revascularisation,

                              Interval Ligation (DRIL)",

                         ifelse(intervent_a2va==11,

                              "Proximalisation of the Arterial Inflow (PAI)",

                         ifelse(intervent_a2va==12,

                              "Revision Using Distal Inflow (RUDI)",

                         ifelse(intervent_a2va==13,

                              "Banding",

                         ifelse(intervent_a2va==14,

                              "CVC insertion",

                         ifelse(intervent_a2va==15,

                              "CVC exchange",

                         ifelse(intervent_a2va==16,

                              "Fibrin sheath removal/disruption",

                         ifelse(intervent_a2va==17,

                              "CVC removal",

                         ifelse(intervent_a2va==18,

                              "Other","No interventions")

                            ))))))))))))))))),

    intervent_a1va = ifelse(intervent_a1va==1,

                               "Open surgical or endovascular

                               creation/placement of AVF/AVG",

                         ifelse(intervent_a1va==2,

                              "Open surgical revision or

                              endovascular intervention

                              of AVG/AVF",

                         ifelse(intervent_a1va==3,

                              "Thrombolysis or thrombectomy of AVG/AVF",

                         ifelse(intervent_a1va==4,

                              "Ligation or resection of Arteriovenous access",

                         ifelse(intervent_a1va==5,

                              "Repair of aneurysm/pseudoaneurysm",

                         ifelse(intervent_a1va==6,

                              "Competing/collateral vein ligation",

                         ifelse(intervent_a1va==7,

                              "Fistulogram (Angiogram) +/- Angioplasty +/-

                              Stenting (including inflow artery,

                              body of AVF/AVG, venous outflow, central vein) ",

                         ifelse(intervent_a1va==8,

                              "Competing/collateral vein embolisation",

                         ifelse(intervent_a1va==9,

                              "Superficialisation/transposition",

                         ifelse(intervent_a1va==10,

                              "Distal Revascularisation,

                              Interval Ligation (DRIL)",

                         ifelse(intervent_a1va==11,

                              "Proximalisation of the Arterial Inflow (PAI)",

                         ifelse(intervent_a1va==12,

                              "Revision Using Distal Inflow (RUDI)",

                         ifelse(intervent_a1va==13,

                              "Banding",

                         ifelse(intervent_a1va==14,

                              "CVC insertion",

                         ifelse(intervent_a1va==15,

                              "CVC exchange",

                         ifelse(intervent_a1va==16,

                              "Fibrin sheath removal/disruption",

                         ifelse(intervent_a1va==17,

                              "CVC removal",

                         ifelse(intervent_a1va==18,

                              "Other", "No interventions")

                            )))))))))))))))))) %>%

  mutate(intervent_a1va = ifelse(is.na(intervent_a1va),

                                 "No interventions",

                                 intervent_a1va),

         intervent_a2va = ifelse(is.na(intervent_a2va),

                                 "No interventions",

                                 intervent_a2va))

  #duration in the study and reasons to exit

  #merge with patient characteristics

  data.end <- read_csv("VALID-StudyEnd_DATA_2020-10-08_1050.csv")

  #screening and enrollment

  data.screen <- read_csv("VALID-ScreeningAndEnrolmen_DATA_2020-10-07_1542.csv")

```

## Clean and merge the datasets

The object *data.pc* contains the patients characteristics at baseline, i.e.,

one row per patient. The dataset set *data.as* has the data collected by

for each assessors.

```{r include=TRUE}

#########################################################################

#REMOVE PATIENTS FROM PATIENTS CHARACTERISTICS THAT DO NOT HAVE        #

#INTERVENTIONS RECORDED                                                 #

#########################################################################

data.pc <- data.pc %>%

            filter(., validid_start %in% dfa2$ID_A2|

                      validid_start %in% dfa1$ID_A1)

#######################################################################

# Recoding the patients characteristics dataset

#######################################################################

    data.pc <- data.pc %>%

                mutate(sex = ifelse(sex_bl==1,"F", "M"),

                 ethnicity =  ifelse(ethnicity_bl==1, "White",

                              ifelse(ethnicity_bl==2, "Black",

                              ifelse(ethnicity_bl==3, "Hispanic",

                              ifelse(ethnicity_bl==4, "Indian",

                              ifelse(ethnicity_bl==5, "Asian",

                              ifelse(ethnicity_bl==6, "American Indian",

                              ifelse(ethnicity_bl==7, "Canadian Aboriginal",

                              ifelse(ethnicity_bl==8, "Aus Aboriginal ",

                              ifelse(ethnicity_bl==9, "Maori",

                              ifelse(ethnicity_bl==10, "Pacific Islander",

                              ifelse(ethnicity_bl==11, "Mixed ethnicity",

                              ifelse(ethnicity_bl==12, "Uncertain",

                              ifelse(ethnicity_bl==13, "Other", NA)

                              )))))))))))),

                comorbidity = ifelse(comorbidity_bl == 1, "Yes", "No"),

                   diabetes = ifelse(typecomor_bl___1==1, "Diabetes",

                              ifelse(typecomor_bl___1==0, "No Diabetes",NA)),

                        cvd = ifelse(cvd_bl___1 == 1 |

                                     cvd_bl___2 == 1 |

                                     cvd_bl___3 == 1, "CVD",

                              ifelse(cvd_bl___1 == 0 &

                                     cvd_bl___2 == 0 &

                                     cvd_bl___3 == 0, "No CVD", NA)),

                       cvd1 = ifelse(cvd_bl___1 == 1, "CVA",

                              ifelse(cvd_bl___1 == 0, "No CVA", NA)),

                       cvd2 = ifelse(cvd_bl___2 == 1, "IHD",

                              ifelse(cvd_bl___2 == 0, "No IHD", NA)),

                       cvd3 = ifelse(cvd_bl___3 == 1, "PVS",

                              ifelse(cvd_bl___3 == 0, "No PVS", NA)),

                   dialysis = ifelse(dialysis_bl ==1, "In-centre",

                              ifelse(dialysis_bl ==2, "Satellite",

                              ifelse(dialysis_bl ==3, "Home haemodialysis", NA)

                              )),

               dialysistime = dialysisyears_bl +

                                dialysismonths_bl/12,

                    access1 = ifelse(access_bl___1 == 1, "Central venous catheters",

                              ifelse(access_bl___1 == 0, "No Central venous catheter", NA)),

                    access2 = ifelse(access_bl___2 == 1, "Arteriovenous graft access",

                              ifelse(access_bl___2 == 0, "No Arteriovenous graft access", NA)),

                    access3 = ifelse(access_bl___3 == 1, "Arteriovenous fistula",

                              ifelse(access_bl___3 == 0, "No Arteriovenous fistula", NA)),

                   location = ifelse(location_bl ==1, "Upper arm",

                              ifelse(location_bl ==2, "Lower arm",

                              ifelse(location_bl ==3, "Leg", NA)

                              )),

                 selfcannu = ifelse(selfcannu_bl ==1, "Rope ladders",

                             ifelse(selfcannu_bl ==2, "Button hole",

                             ifelse(selfcannu_bl ==3, "Other", NA)

                              ))

                )

#######################################################################

#######################################################################

# Number of interventions by assessor 1

#######################################################################

ninterv.a1 <- length(unique(dfa1$ID_A1))

#######################################################################

# Number of interventions by assessor 3

#######################################################################

ninterv.a2 <- length(unique(dfa2$ID_A2))

#######################################################################

#duration in the study and reasons to exit dataset

#######################################################################

   data.end  <- data.end %>% mutate(last_date = as.Date(

                                ifelse(date_exit_end =="",

                                          date_study_end,

                                             date_exit_end),

                                             format="%d/%m/%y"))

  #merge date enrolled with date starting the study

  data.end <- left_join(data.end,

                        data.screen %>%

                          select(c("validid_start", "date_enrol_scr")),

                        by = "validid_start")

  #time in the study

  data.end <- data.end %>%

                  mutate(time_instudy = as.numeric(last_date -

                        as.Date(date_enrol_scr, format="%d/%m/%y")))

  #merge time in the study with patients characteristics

  data.pc <- left_join(data.pc,

                    data.end %>%

                      select(c("validid_start", "time_instudy", "reason_end")),

                    by="validid_start")

```

# Main Statistical Analysis - VALID study

## Descriptive statistics - baseline characteristcs

There were `r table(data.pc$patient_characteristics_complete)`

patients with complete information in patients' characteristics dataset.

Patients characteristics are displayed in table 1. Table 2 presents the

characteristics stratfied by participant centre.

```{r table1, include=TRUE}

#####Table 1

  pat.char <- names(data.pc) [-c(1:5, 11,12,13,33,35)]

  pat.char <- c("age_bl", "sex", "height_bl",

                "weight_bl", "bmi_bl",  "ethnicity",

                "comorbidity",

                "diabetes", "cvd", "cvd1", "cvd2", "cvd3",

                "dialysis", "dialysistime",

                "access1", "access2", "access3", "time_instudy")

 CreateTableOne(vars=pat.char,

                data=data.pc) %>%

  print(nonnormal = c("dialysistime", "time_instudy"),

             printToggle = FALSE,

             noSpaces = TRUE) %>%

  kable(p1, format = "html",

        booktabs = T,

        caption = "Patients' characteristics") %>%

  kable_styling()

```

```{r table 2}

 #####Table 2

CreateTableOne(vars=pat.char,  strata="redcap_data_access_group",

                data=data.pc, test=FALSE)  %>%

 print(nonnormal = c("dialysistime", "time_instudy"),

             printToggle = FALSE,

             noSpaces = TRUE) %>%

  kable(format = "html",

        booktabs = T,

        caption = "Patients' characteristics by Centre") %>%

    kable_styling()

```

## Sensitivity and Specificity of events identifcation

In this section we analyse the sensitivity, specificity and accuracy of the

procedures dates.

We are taking assessor 2 as the *reference standard*. The table below presents

the raw (no clustering taken into account) specificity and sensitivity

```{r SensSpec, echo=FALSE}

  #Raw Sensitivity and Specificity

  crosstable(data.as, c(hasinterventions_a1va),

             by = hasintervention_a2va,

             percent_digits=0,

           percent_pattern="{n} ({p_col})") %>%

  as_flextable(keep_id=TRUE)

  naive.sens.spec <- data.as %>%

    select(c(hasinterventions_a1va,

             hasintervention_a2va)) %>%

    table() %>% epi.tests()

```

The raw sensitivity (correctly identified interventions) is

`r round(naive.sens.spec$detail$se,2)[1]*100`%  and the raw specificity

(correctly identified non interventions) is

`r round(naive.sens.spec$detail$sp,2)[1]*100`%.  The proportion of correctly

collected data is `r round(naive.sens.spec$detail$diag.ac,2)[1]*100`%.

We now compute the sensitivity and specificity based on a logistic regression

fitted with a gee to take into account the cluster per patient and per centre.

Given that the majority of the centres has one assessor 1 and one assessor 2,

the clustering per centre is similar to the clustering by assessor.

The table below presents the validity statistics with

the respective 95% confidence intervals

```{r SensSpec_model, echo=FALSE}

#need to sort by ID for the gee

data.as <- arrange(data.as,ID)

#fits a logistic regression with GEE

model.sens.spec <- geeglm(hasinterventions_a1va~hasintervention_a2va,

                     data=data.as, id=ID,

                     family=binomial,

                     corstr="independence")

#Predicted probabilities (not running due to small numbers)

  table.sens.spec <- ggpredict(model.sens.spec,

                       terms = "hasintervention_a2va")

  table.sens.spec[1,1] <- "1-specificity"

  table.sens.spec[2,1] <- "sensitivity"

   kable(table.sens.spec, format = "html",

          booktabs = T,

          caption = "Patients' characteristics", digits = 2) %>%

      kable_styling()

```

```{table 2}

tibble(labels = c("unadjusted sensitivity", "unadjusted specificity",

                  "patient ajdusted sensitivity",

                  "patient ajdusted specificity"),

       value=c(naive.sens[1],

             naive.spec[1],

             table.sens.spec[2,2],

             1-table.sens.spec[1,2]) %>%

           round(2) ,

             ConfidenceInterval95Lower = c(naive.sens[2],

                                           naive.spec[2],

                                           table.sens.spec[2,4],

                                           1-table.sens.spec[1,5])%>%

                                         round(2),

             ConfidenceInterval95Upper = c(naive.sens[3],

                                           naive.spec[3],

                                           table.sens.spec[2,5],

                                           1-table.sens.spec[1,4]) %>%

                                         round(2)

       ) %>%

   kable(format = "html",

          booktabs = T,

          caption = "Patients' characteristics", digits = 2) %>%

      kable_styling()

```

## Number of interventions

Table \ref{tab:totinterventions} show the agreement on the number of interventions per

patient. Notice, that this not take into account the date or type of

interventions recorded.  If assessor 1 records 3 interventions for patient A and

assessor 2 also records 3 interventions but of different type and different

dates, this is counted as perfect agreement.

The weighted Kappa statistics and 95% confidence interval associated with

table \ref{tab:totinterventions} is provided below.  The weights

used are quadratic weights (default).  The ICC would be an alternative measure

of agreement but given the low number of discrete values, we opted for the

weighted kappa for ordinal variables. In any case, the ICC value is very similar

to the kappa, as expected.

```{r totinterventions, echo=FALSE}

#nr pat with 0,1,2,3,4,... interventions by a1

  #number of per patient by assessors 1 and 2

  n.interv <- ddply(data.as,

                  .(ID_A1),

                  summarize,

                  `Numero VA interventions assessor 1` =

                      sum(hasinterventions_a1va),

                  `Numero VA interventions assessor 2` =

                      sum(hasintervention_a2va)) %>%

            select(-"ID_A1")

  #table

  n.interv %>%

  table() %>%

  kable(format = "html", booktabs = T,

        caption = "Number of patients with 1, 2, 3, ...

        interventions recorded by assessor 1 (a1) and 2 (a2)")

  #agreement between the number of VA interventions

  #between assessors 1 and 2 measure by weighted kappa

  #using a squared weight

  n.interv %>%  kappa2( weight="squared")

  #95% Confidence interval for the kappa

  print("Confidence interval for kappa")

  kappa2.bootstrap.ci(n.interv,

                            nbt=1000,

                            wght = "squared") %>%

    round(2)*100

```

Each patient had in average

`r round(sum(data.as$hasintervention_a2va)/(sum(data.pc$time_instudy)/365.25),2)`

interventions per year.

## Agreement on the type of interventions

Distribution of the type of interventions for each assessor

```{r type-assess, echo=FALSE}

data.as %>% select(c("intervent_a1va", "intervent_a2va")) %>%

   dplyr::rename(`Assessor 1` = intervent_a1va) %>%

     dplyr::rename(`Assessor 2` = intervent_a2va) %>%

  gather(., Assessor, Intervention,

         `Assessor 1`:`Assessor 2`,

         factor_key=TRUE) %>%

  CreateTableOne(vars="Intervention",

                 strata="Assessor",

                data=., test=FALSE) %>%

 print(printToggle = FALSE,

             noSpaces = TRUE) %>%

  kable(format = "html",

        booktabs = T,

        caption = "Type of interventions") %>%

    kable_styling()

```

The agreement between assessors 1 and 2 in terms of type of interventions

is presented in table. "No intervention" was considered as a category

of intervention. Columns refer to assessor 2 and rows to assessor 1.

The kappa statistics is also provided.

```{r agreement, echo=FALSE}

  #type on intervention by assessors 1 and 2

  type.intervention <-  data.as %>%

    select(c("intervent_a1va", "intervent_a2va"))

  #table

  type.intervention %>%

  table() %>%

  kable(format = "html", booktabs = T,

        caption = "Number of patients with 1, 2, 3, ...

        interventions recorded by assessor 1 (a1) and 2 (a2)") %>%

    kable_styling()

  #agreement between the type of VA interventions

  #between assessors 1 and 2 measure by simpl kappa

  type.intervention %>%  kappa2()

  #95% Confidence interval for the kappa

  print("Confidence interval for kappa")

  kappa2.bootstrap.ci(type.intervention,

                            nbt=1000,

                            wght = "unweighted") %>%

    round(2)*100

```
